# Impaired myogenic development, differentiation and function in hESC-derived SMA myoblasts and myotubes

**DOI:** 10.1371/journal.pone.0205589

**Published:** 2018-10-10

**Authors:** Nicole Hellbach, Suzanne Peterson, Daniel Haehnke, Aditi Shankar, Samuel LaBarge, Cullen Pivaroff, Stefanie Saenger, Carolin Thomas, Kathleen McCarthy, Martin Ebeling, Monica Hayhurst Bennett, Uli Schmidt, Friedrich Metzger

**Affiliations:** 1 Roche Innovation Center Basel, Roche pRED, Pharma Research & Early Development, Grenzacherstrasse, Basel, Switzerland; 2 Genea Biocells, North Torrey Pines Rd, San Diego, CA, United States of America; University of Edinburgh, UNITED KINGDOM

## Abstract

Spinal muscular atrophy (SMA) is a severe genetic disorder that manifests in progressive neuromuscular degeneration. SMA originates from loss-of-function mutations of the *SMN1* (Survival of Motor Neuron 1) gene. Recent evidence has implicated peripheral deficits, especially in skeletal muscle, as key contributors to disease progression in SMA. In this study we generated myogenic cells from two SMA-affected human embryonic stem cell (hESC) lines with deletion of *SMN1* bearing two copies of the *SMN2* gene and recapitulating the molecular phenotype of Type 1 SMA. We characterized myoblasts and myotubes by comparing them to two unaffected, control hESC lines and demonstrate that SMA myoblasts and myotubes showed altered expression of various myogenic markers, which translated into an impaired *in vitro* myogenic maturation and development process. Additionally, we provide evidence that these *SMN1* deficient cells display functional deficits in cholinergic calcium signaling response, glycolysis and oxidative phosphorylation. Our data describe a novel human myogenic SMA model that might be used for interrogating the effect of SMN depletion during skeletal muscle development, and as model to investigate biological mechanisms targeting myogenic differentiation, mitochondrial respiration and calcium signaling processes in SMA muscle cells.

## Introduction

Spinal Muscular Atrophy (SMA) is an autosomal-recessive genetic neuromuscular disease characterized by a progressive loss of motor neurons in the anterior horn of the spinal cord leading to proximal muscle weakness and paralysis [1,2]. SMA is one of the most devastating neurological diseases in childhood representing the number one cause of death related to genetic dysfunction in infants and toddlers [3]. SMA is caused by loss of function mutation of the *SMN1* gene leading to deficiency of survival motor neuron (SMN) protein [4]. This leads to the death of anterior horn motor neurons in the spinal cord and system-wide muscle atrophy resulting in progressive weakness and disability [5]. In humans the highly homologous *SMN2* gene can partly compensate for the loss of *SMN1*. However, *SMN2* has one transcriptionally silent point mutation (SMN2 c.840 C>T) in the coding sequence, which is of major importance for the functionality of the resulting protein. Specifically, it modifies the splicing of *SMN2* mRNA, leading to exclusion of exon 7 (SMNΔ7) in the majority of the transcripts. The lack of exon 7 furnishes an unstable protein [6–8]. SMA patients therefore express reduced levels of full length SMN protein and exhibit varying disease phenotypes, dependent on *SMN2* copy number which can vary from 0 to 8 copies [9–16].

SMA was traditionally classified as a disease of the lower motor neurons, which explains the investigational focus on the intrinsic deficits in motor neurons [3,17] and the clinical manifestations linked to the dysfunction of motor neurons and the neuromuscular junction. However, it is becoming increasingly clear that additional cell and tissue types may play a role in the disease pathology. Accumulating evidence has shown SMN depletion to effect other CNS compartments, including astrocytes, microglia, and neurons [18–21], as well as peripheral tissues, contributing to the progression of disease in SMA patients and in pre-clinical models [22,23].

Of particular note, skeletal muscle appears to be an important tissue that contributes to the pathophysiology of SMA [24–26]. Motor neurons and skeletal muscle are closely linked and rely upon continued association to maintain healthy neuromuscular junctions. Whereas neuronal dysfunction can contribute to muscle atrophy, there is mounting evidence that intrinsic abnormalities in the SMA skeletal muscle cells could play a primary role in this disease [23,27]. In both mice and flies, high levels of Smn are reported to localize to the myofiber, suggesting a muscle-specific function [28,29]. Additional evidence suggests that high levels of Smn are critical for the survival of myogenic (muscle-forming) cells. For example, cultured myogenic cells harboring a deletion of Smn exon 7 and producing a rapidly degrading truncated form of the protein showed increased cell death through a non-apoptotic process [30]. Furthermore, reduction of Smn in C2C12 cells caused reduced proliferation, defects in myoblast fusion, and malformed myotubes [31]. Studies conducted in mouse models strongly suggested an additional role for Smn in muscle development and maintenance. Analysis of skeletal muscle tissue in various severe SMA mouse models (S*mn*^−/−^;*SMN2*^*+/+*^; S*mn*^−/−^;*SMN2*^*+/+*^*; SMN2Δ7*, *SMN*^*C/C*^*)* demonstrated reduced size of muscle fibers [32]. Interestingly, treatment of *SMNΔ7* mice with scAAV9-SMN could improve the muscular phenotype, but even early treatment at P2 could not completely restore the muscle pathology [33]. Selectively abolishing Smn expression in muscle tissue results in a prominent dystrophic phenotype, characterized by myofiber necrosis associated with destabilization of sarcolemma components [26]. Interestingly, this phenotype could be improved by maintaining Smn expression in myogenic precursors, with depletion of Smn being localized to multinucleated myotubes[30]. Moreover, SMN-deficient myotubes cultured from SMA patient biopsies are smaller in size compared to myotubes prepared from normal patient biopsy tissue [34].

There are two challenging aspects of studying a cell autonomous defect in human SMA myogenic cells which can be overcome using human pluripotent stem cell (hPSC), including either human embryonic stem cell (hESC) or human induced pluripotent stem cells (hiPSC), derived myogenic cells. First, acquiring primary muscle biopsy material requires invasive, painful, and costly procedures which provide a high burden to the patients and limited quantities of research material. Second, myoblasts derived from patients have likely been exposed to atrophying motor neurons and muscle which can amplify or mask cell autonomous defects. In contrast, genetically affected hESC, which are considered the “gold standard,” have proliferative and differentiation properties that make them an inexhaustible source for research material without patient burden. Myogenic cells derived from hPSCs allows the monitoring of a cell autonomous differentiation process, without the confounding effects of motor neuron contact at the neuromuscular junction.

In this study, we generated myogenic cells from two SMA-affected hESC lines bearing two copies of the *SMN2* gene and compared them with two unaffected control hESC lines. We demonstrate an impairment in myogenic marker expression in hESC-derived myoblasts and myotubes. It translates to a respective alteration in myogenic maturation that confirms many of the findings using mouse cells and tissue. Furthermore, we also describe metabolic changes in SMA myogenic cells that lead to impaired glycolysis, oxidative phosphorylation, and mitochondrial respiration.

## Material and methods

### Cell culture

Healthy control hESC lines were derived and banked by Genea Biocells (Australia) in compliance with international guidelines, including the U.S. National Acadamies’ 2008 Guidelines for Human Embryonic Stem Cell Research [35,36]. Ethics approval for the project (‘the possibility that karyotyped embryonic cells derived from non-viable “investigation” embryos could be developed into stem cell lines’) was obtained from the Genea Ethics Committee on 13 September 2005 under the Ethical Guidelines on the Use of Assisted Reproductive Technology in Clinical Practice and Research (ART guidelines, 2004) and the National Statement on Ethical Conduct in Human Research. Karyotyped embryo cells were fully consented for development of stem cells by all responsible people through an informed consent process (signed de-identified consent form can be provided upon request). Both lines are commercially available, product numbers G016-PSC and G019-PSC. SMA-affected hESC lines were derived and provided by Dr. Rachel Eiges (Shaare Zedek Medical Center, Israel). The Bioethics Advisory Committee of the Israel Academy of Sciences and Humanities reviewed and approved SMA cell line development. Informed, written consent was given by participants to donate affected embryos for the purpose of derivation of embryonic stem cell lines.

Myoblasts were generated as described from SMA and unaffected hESCs [37]. Briefly, myoblasts were generated from ESCs by plating hESCs at low density on collagen1 coated plates, exposing them to skeletal muscle media for approximately 10 days, and finally culturing cells in myoblast medium for 4–5 days. Cryopreserved stocks of Genea016 (Healthy_A), Genea019 (Healthy_B), Genea102 (SMA_A, SMA Type 1), or Genea103 (SMA_B, SMA Type 1) myoblasts were thawed in a 37°C water bath and resuspended in Stage 2B medium (#SKM-02, Genea Biocells) or Skeletal Muscle Cell Growth Medium (#C23060, PromoCell). Thawed cells were then centrifuged for 5 min at 400 x*g*, supernatant was aspirated, and the cell pellet was resuspended in 1 mL of the appropriate medium. Cells plated as myoblasts were counted as cell culture Day 0.

For differentiation to myotubes, myoblasts were cultured to ~90% confluency in Skeletal Muscle Cell Growth Medium and then switched to DMEM (#31966–021, Life Technologies) + 5% horse serum (#26050–070, Life Technologies). Alternatively, cells were cultured in Stage 2 medium (#SKM-02, Genea Biocells) for 4 days and then switched to Stage 3 medium (#SKM-03, Genea Biocells) for an additional 3 days to facilitate myotube differentiation.

For expansion purposes, cells were grown in non-coated flasks (#353112, #353135, Corning), detached using TrypLE Express (#12604–013, Life Technologies) and seeded at the required cell density. Cells were counted with Trypan Blue Solution (#056580–1001, Roche Applied Science) using Cedex XS Analyzer (#05926432001, Roche Applied Science) or with Acridine Orange/Propidium Iodide stain (#F23001, Logos Biosystems) and a Lunastem Automated Fluorescence Cell Counter (#L30001, Logos Biosystems).

For the experiments, cells were seeded on collagen I-coated plates (#356500, #356407, Corning) at 30000 cells/cm^2^ in Stage 2B medium or skeletal muscle cell growth medium and incubated at 37°C in 5% CO_2_. Brightfield microscopy pictures were taken using Eclipse TS100 and DS-Fi2 camera (both Nikon).

### Determination of SMN copy number

The cell lines were sequenced by Sanger Sequencing and SMN1/SMN2 copy number was determined by Dr. Matthew Butchbach, Nemours Biomedical Research and their Biomolecular Core Lab. Briefly, cell lines were tested for SMA by genomic PCR (ddPCR) and Sanger sequencing [10]. Cells were analyzed for single nucleotide polymorphisms (SNP) within SMN2 by Sanger sequencing and showed no variant SNPs [38,39]. SMN1 and SMN2 copy number was measured using digital droplet PCR (Bio-Rad) according to manufacturer`s instructions [10].

### qRT-PCR

Cell were lysed using TaqMan Gene Expression Cells-to-CT^TM^ Kit (#AM1729; Life Technologies) according to manufacturer`s instructions. Briefly, cells were washed with cold DPBS (#140190–094, Life Technologies) and lysed with 50μl/cm^2^ Lysis Buffer + 1% DNase I. After 5 min incubation at RT, 5μl/cm^2^ Stop Solution was applied and incubated for 2 min at RT. 2μl sample and 8μl RT-qPCR master mix (5μl 2x RT-PCR buffer and 0.4μl 25xRT/PCR enzyme (both #AM1729, Life Technologies), 1.6μl H_2_O (#4388514, Life Technologies) and 0.5μl each primer (for details see materials) was added into a LightCylcer 480 Multiwell 384 Plate (#0521755001, Roche Applied Science), sealed with foil (#04729757001, Roche Applied Science) and run in a LightCycler 480 Instrument II (Roche Applied Science) using LightCycler 480 software (Roche Applied Science). Reactions were run in triplicate, Cp values were determined with LightCycler 480 software, normalized to healthy cells and calculated using the 2^-ΔΔCp^ method. The following primers/probes were used (5´- 3´):

SMN2 FL forward GCTCACATTCCTTAAATTAAGGAGAAA;

SMN2 _7 forward: TGGCTATCATACTGGCTATTATATGGAA;

SMN2 reverse TCCAGATCTGTCTGATCGTTTCTT;

SMN2 probe: FAM-CTGGCATAGAGCAGCACTAAATGACACCAC-BHQ-1

(all purchased from microsynth); Human GAPDH Endogenous Control (#4326317E; Life Technologies); MYOD (#Hs02330075 g1, Life Technologies); MYOG (#Hs0107223 m1, Life Technologies), CHRNA1 (#HS001755478_m1, Life Technologies); CHRNG (#Hs00183228_m1, Life Technologies) 5´-conjugated FAM and 3´-conjugated MGB-NFQ (#4351370, Life Technologies).

### qPCR

Medium was removed and cells lysed using AllPrep DNA/RNA Mini Kit (#80204, Qiagen) or AllPrep DNA/RNA 96 Kit (#80311, Qiagen) according to the manufacturer`s instructions. DNA levels were measured using Nanodrop 1000 (ThermoFisher Scientific). 2μl of sample (5ng/μl) was mixed with 8μl of PCR master mix (2x LightCycler 480 Probe Master (#04707494001, Roche Applied Science), 0.5μl each primer (see materials)), 10U/μl HaeIII (#ER0151, ThermoFisher Scientific)), added into a LightCycler 480 Multiwell 384 Plate (#0521755001, Roche Applied Science) sealed with foil (#04729757001, Roche Applied Science) and run in a LightCycler 480 Instrument II (Roche Applied Science) using LightCycler 480 software (Roche Applied Science). Reactions were run in triplicate, Cp values were determined with LightCycler 480 software, normalized to healthy cells and calculated using the 2x2^(nDNA-mtDNA)^ method (Gonzales-Hunt et al., 2016). The following primers/probe combination was used: RPP30 conjugated with FAM (#dHsaCP2500313, Bio-Rad), ND1 conjugated HEX (#dHsaCNS777311275, Bio-Rad), B2M conjugated FAM (#dHsaCNS334344787, Bio-Rad), ND5 conjugated with HEX (#dHsaCNS548010382, Bio-Rad)

### Immunoblotting

Briefly, for western blotting, cell lysis was performed by removing medium and directly applying hot 2x Laemmli Sample Buffer (#161–0737, Bio-Rad) and 0.2M dithiothreitol (DTT, #D0632, Sigma-Aldrich) for 10 min at 95°C. 10μl of sample were loaded on Bis-Tris Gels (4%-12%, #345–0125, Bio-Rad) or Tris-Acetate Gels (3%-8%, #345–0131, Bio-Rad) and separated using NuPAGE MES SDS Running Buffer (#NP0002, Life Technologies) or NuPAGE Tris-Acetate SDS Running Buffer (#LA0041, Life Technologies) both diluted 1x with H_2_O. Proteins were transferred using the iBlot system (#IB1001, Life Technologies) to a nitrocellulose membrane (#RPN303E, GE Healthcare Life Science) either for 5 min (Bis-Tris) or 7 min (Tris-Acetate) according to manufacturer`s instructions. Membranes were blocked and then incubated with blocking buffer (1:1 Blocking Buffer (#927–40000, Li-Cor) and PBS (#11666789001, Roche Applied Science) + 0.5% Tween20 (#93773, Sigma-Aldrich) as well as diluted primary antibody at 4°C o/n. The following antibodies were used: β-actin (#sc-1615, Santa Cruz, 1:20000, internal control); Desmin (#sc-70961, Santa Cruz, 1:5000), SMN (#610647, BD BioSciences, 1:5000). After washing with PBST at RT, membranes were incubated with secondary antibody (donkey anti-mouse (#610-730-124, Rockland, 1:2000), donkey anti-goat (#605-732-125, Rockland, 1:10000)) for 1h at RT. Stained membranes were scanned with an Odyssey Infrared Imager (#9120, Li-Cor) and values normalized to internal control.

For capillary western blotting, cells were lysed for 5 min using 1x RIPA buffer (#R0278, Sigma-Aldrich) with Phosphatase Inhibitor Cocktail (#04906845001, Roche Applied Science) and complete Protease Inhibitor Cocktail (#04693124001, Roche Applied Science) or RIPA supplemented with Halt Protease inhibitor cocktail (#20–188, Millipore) and Halt Phosphatase inhibitor cocktail (#1861277, ThermoFisher Scientific). Total protein in the supernatant was quantified by bicinchoninic acid assay (#23225, ThermoFisher Scientific) or Nanodrop 1000. Samples were prepared according to manufacturer`s instructions using Core Kit for Sally (#CBS301, ProteinSimple) and capillary western blot was performed using Sally or Wes Simple Western (ProteinSimple). Following antibodies were used; α-tubulin (#2144, Cell Signaling, 1:50, internal control), MYH1/2/4/6 (#sc-32732, Santa Cruz,1:200), SMN (#610647, BD BioSciences, 1:50), HSP90 (#ab58950, Abcam, 1:50), donkey anti-mouse (#042–205, ProteinSimple), goat anti-rabbit (#042–206, ProteinSimple). Data were analyzed using Compass Software (ProteinSimple) following the manufacturer’s protocol using α-tubulin as internal standard.

### Immunocytochemistry

Cells were cultured as described above and plates were washed in PBS and fixed on the indicated day after seeding in 10% Formalin or 4% PFA for 10 min. Fixed cells were incubated for 1h using a blocking solution of 5% BSA, 0.3% Triton X-100 or 2% horse serum, 0.1% Tween in PBS. Mouse anti-MYOD (#554130, BD, clone 5.8A, 1:200),mouse anti-PAX3 (Pax3, Developmental Studies Hybridoma Bank, 1:200), mouse anti-MYH1/2/4/6 (#sc-32732, Santa Cruz, 1:200), mouse anti-MYOG (#ab1825, abcam, 1:200), mouse anti-TNT (#T6277, Sigma-Aldrich, 1:200), anti-MYH (MF 20, DSHB, 1:1000) and Ki67 (#9129S, Cell Signaling Technologies, 1:1000) were diluted in the blocking solution and incubated for 1 h at RT. Cells were then washed with PBS three times. Secondary antibodies, anti-mouse IgG1, anti-mouse IgG2a and anti-rabbit IgG1 conjugated with Alexa Fluors (#A211-31, A211-25, R37117, ThermoFisher Scientific) were used at 1:1000 for 1 hr at RT. Cells were then washed 3X with PBS and then the nuclei were counterstained with Hoechst 33342 (#62249, Thermo Scientific 1:5000). Immunolabeled plates were imaged using an INCell Analyzer 6000 (GE Healthcare) high content imaging system and fluorescence intensity was quantitated using the Developer Toolbox software version 1.9.3. The fluorescence intensity of treated cells was compared to DMSO treated cells using Microsoft Excel software.

### NanoString

Cells were cultured as described and washed once in PBS. Cell lysates were then harvested in 10μl of iScript RT-qPCR Sample Preparation Reagent (BioRad) per well of a 96 well plate. Samples were immediately frozen at -80°C. NanoString analysis was performed as recommended by the manufacturer. Briefly, 5μl of lysate was mixed with 8μl of hybridization buffer containing reporter probes from our custom SMA code set containing 71 muscle-related genes as illustrated in Table 1 (NanoString Technologies, Inc.). From there, 2μl of capture probes were added and the samples were hybridized for 16 hours at 65°C. The next day, samples were diluted in 16μl of water and added to the NanoString cartridge. The cartridge was processed using a NanoString SPRINT machine and data was analyzed using N-Solver software (NanoString Technologies, Inc.). Fold-change is depicted.

**Table 1:** Myogenic marker expression changes in SMA myoblasts. RNA expression levels were analyzed by a NanoString array containing 71 genes of known to be involved in skeletal muscle development and function. Data were used for the pathway analysis in Fig 3. Solver software (NanoString Technologies, Inc.). Fold-change was depicted. Eleven transcripts that belong to the “Muscle_contraction” network from REACTOME are highlighted in yellow. This network is significantly down-regulated in SMA myoblasts as a whole.

Gene	Fold change	Gene	Fold change	Gene	Fold change	Gene	Fold change
IGF1	4,287	ASCC1	0,405	PLS3	-0,248	TNF	-0,669
HOXA9	3,231	IGHMBP2	0,400	SOD1	-0,253	BAX	-0,678
NFIX	3,185	MEF2A	0,334	MYL3	-0,276	SMN1	-0,712
MYF6	1,921	IL6	0,300	UBA1	-0,281	BMP4	-0,720
SQSTM1	1,619	MAP2K2	0,268	CASP3	-0,286	ZEB2	-0,845
UCHL1	1,177	TRIP4	0,267	MYL1	-0,349	TNNI1	-0,868
CNTN1	1,146	CHEK1	0,251	COIL	-0,350	DMD	-0,930
NAIP	1,086	CREB1	0,118	GATA6	-0,538	SMAD3	-0,953
PAX3	0,757	CUL5	0,091	PTGS2	-0,561	MAP2K1	-0,955
ACTA1	0,740	CAMK2G	0,079	DAG1	-0,611	DLL1	-0,982
CAPN3	0,739	DYNC1H1	0,010	CAV1	-0,635	MYOD1	-1,171
BCL2	0,653	NAE1	-0,058	CAMK2A	-0,638	TNNI2	-1,321
IGF2BP1	0,632	PAX7	-0,100	AKT1	-0,650	SERPINE1	-1,405
VAPB	0,538	TARDBP	-0,105	TWIST2	-0,658	MYH3	-1,579
CKM	0,503	ACVR1B	-0,145	MYH2	-0,661	DES	-1,631
MIB1	0,464	TCF4	-0,190	MYH8	-0,668	CAV3	-2,252
ZPR1	0,434	MYOG	-0,230	MYF5	-0,669	MYH7	-3,218
MSTN	0,431	NEDD8	-0,234	MYH4	-0,669		

### Network analysis

Direct pairwise interactions for the entire list of genes of the NanoString assay was extracted for their pairwise interaction documented in the MetaBase (www.metabase.com) database, focusing on direct interactions, excluding cleavage events and ubiquitination events. The Pathway Commons collection of annotated networks (www.pathwaycommons.org) was searched for networks that were significantly over-represented in the NanoString gene list based on Fisher´s exact test (hypergeometric distribution).

### Seahorse analysis of cell metabolism

Myoblasts were seeded as described above in XFp-formatted plates (#103022–100, Agilent) at approximately 10,000 cells/well in Stage 2B medium. The following day, the Stage 2B medium was changed. One day prior to analysis, sensor cartridges were hydrated with calibration buffer and placed in a 37°C incubator without CO_2_. After 3 days, the medium was changed to Seahorse XF Base Medium (pH 7.4) containing 4mM Glutamine, 5.5mM Glucose, 2mM Pyruvate, and 5mM HEPES. Cells were placed in a 37°C incubator without CO_2_ for 1 hour prior to analysis using the Mito Stress Test (#103010–100, Agilent) or the Glycolytic Rate Assay (#103346–100, Agilent). For the Mito Stress Test, oligomycin was added at 3uM, the FCCP concentration used was 2uM and the Antimycin A/Rotenone concentration was 0.5uM. For the Glycolytic Rate Assay, 0.5uM Rotenone/Antimycin A and then 50mM 2-Deoxyglucose was added to the cells. After Seahorse analysis, cells were fixed, stained with DAPI, imaged on an IN-Cell Analyzer 6000 (GE Healthcare) and nuclei counts were generated using the Developer Toolbox software. Seahorse results were normalized to cell counts. Normalized Seahorse data were analyzed using Wave (software for XFp Analyzer, Agilent), and statistical calculations and graphical representations were created as well as Prism (Graphpad) software.

### ATP levels

Cells were cultured as described above to generate myoblasts and myotubes. Twenty-four hours before harvest, cells were treated with Cyclosporin A (#AAJ6319103, Fisher Scientific) at 5μM. Two hours prior to harvest, cells were treated with Oligomycin A at 10μg/ml. ATP levels were analyzed using the Cell Titer Glo kit (#G7571, Promega) according to the manufacturer’s instructions. Luminescence was measured using a SpectraMax i3X plate reader (Molecular Devices).

### Assessment of intracellular Ca^2+^ concentrations ('calcium imaging')

Intracellular Ca^2+^ concentrations were measured with a fluorometric assay. In detail, cells were plated on pre-coated collagen-I black-clear 96-well plates (#356649; Corning) and differentiated for 3 days after they reached nearly confluency. Medium was removed and cells were incubated for 30min at 37°C with 100 μl loading buffer (consisting of imaging buffer (ultrapure H2O (#10977035; Life Technologies), 1x HBSS (#14065–049; Life Technologies), 5mM HEPES (#15630–056; Life Technologies)) and 2.5 μM Fluo-4 AM (#F14202; Life Technologies, in DMSO). After twice washing with 250μl imaging buffer, cells were incubated for 10min at 31°C in the dark with 100μl of imaging buffer. The fluorescent signal was measured at RT with an FDSS 7000 Fluorometric Plate Imaging Reader (Hamamatsu) and FDSS 3000/6000/7000 software (Hamamatsu). Exposure time was 200 ms and automatic imaging sensitivity. Baseline levels were measured for at least 2 min. Treatment with 25 μL 5x solution carbamoylcholine chloride (#C4382; Sigma-Aldrich, diluted in imaging buffer) was performed and samples measured. 4-Br-A23187 (#B7272; Sigma-Aldrich, 1μM final concentration) was added at the end of the experiment as 25 μl of 6x solution per well. Carbamoylcholine chloride response was defined as the subtraction of peak fluorescent signal within 15s and average of fluorescence within 5s before carbamoylcholine chloride addition. Carbamoylcholine chloride response was then normalized by the 4-Br-A23187 response. The 4-Br-A23187 response was also defined as the peak fluorescence after 4-Br-A23187 addition subtracted by the average fluorescence within 5s prior carbamoylcholine chloride treatment. Final normalization to the individual maximal carbamoylcholine chloride within an experiment was performed.

### Statistical analysis

Statistical analyses were performed using unpaired two-tailed Student’s t-test in Prism 7 (La Jolla, CA) if not noted otherwise in the legend. For group comparisons, one-way ANOVA followed by Tukey's multiple comparisons test was used. For time-dependent analyses, two-way ANOVA followed by Sidak's multiple comparisons test was used. Data were considered significant at p<0.05.

## Results

### Myoblasts from SMA patients express molecular phenotypes of SMA

**Fig 1:**
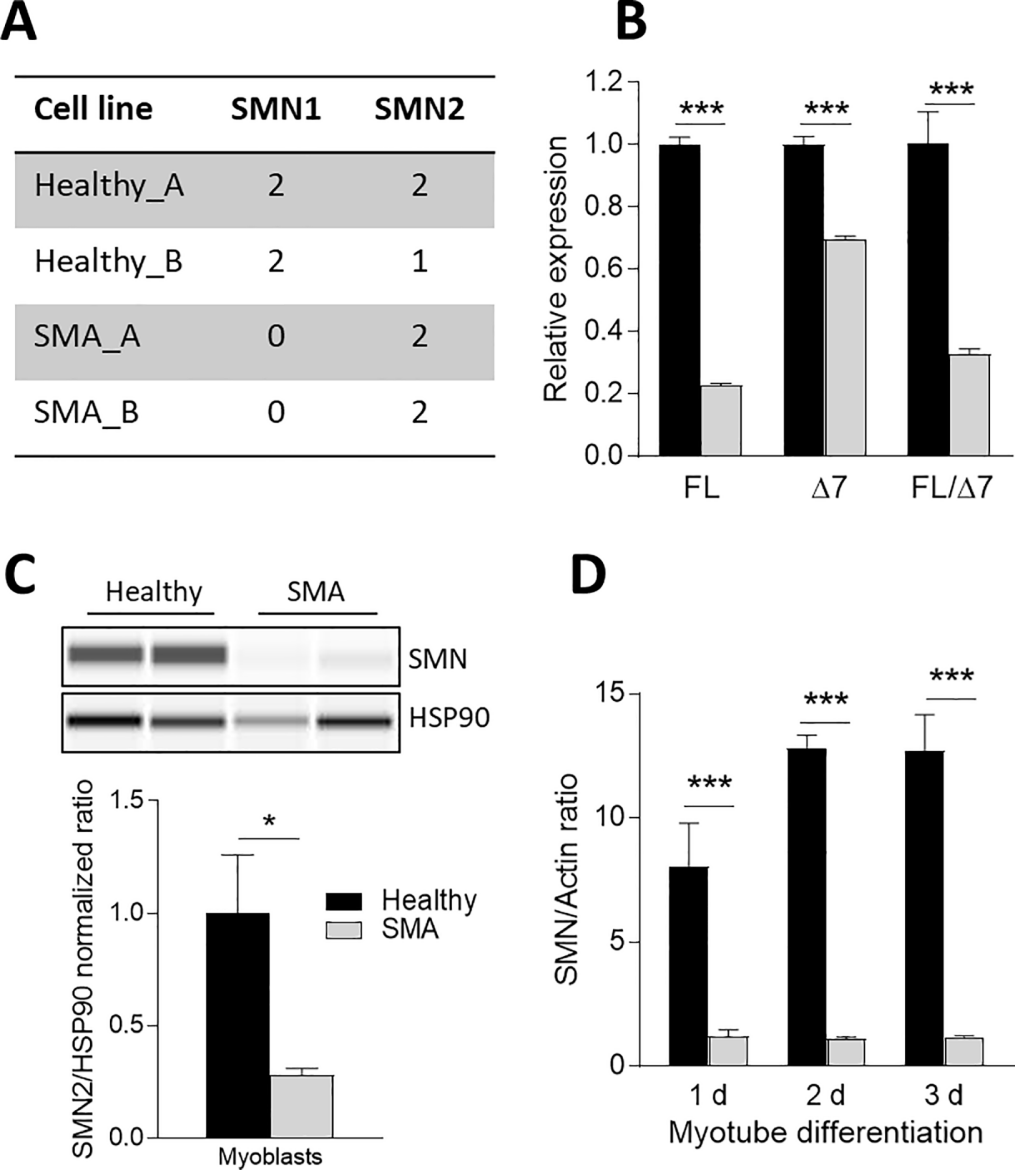
Impaired SMN2 splicing and reduced SMN protein in myoblasts from SMA patients. Molecular analysis of the two hESC unaffected and the two hESC SMA Type 1 lines revealed no SMN1 expression in the genomic PCR analysis, but 2 SMN2 copy numbers (A). *SMN2FL* (FL) and *SMNΔ7* (Δ7) transcripts were significantly reduced (B). SMN protein levels in SMA Type 1 remained low compared to unaffected myoblasts (C), and were stable over 3 days after change to differentiation medium (D). Data represented as mean ± SEM of n = 6–12 individual samples and normalized to unaffected and endogenous controls (B: GAPDH, C: HSP90, D: Actin). *: p < 0.05; **: p < 0.01; ***: p < 0.001, two-tailed unpaired Student's t-test.

To investigate whether myogenic differentiation is altered in SMA we derived myogenic cells, including myoblasts and myotubes from SMA patients and unaffected controls hESCs using the protocol previously described in Caron et al. (2016) [37]. First, we measured the copy numbers of *SMN1* and *SMN2* from genomic DNA isolated from hESC derived myoblasts from 2 healthy and 2 SMA lines using digital droplet PCR [10]. The ddPCR assay focused on exon 7, which contains the single nucleotide variant which functionally distinguishes *SMN1* from *SMN2*. Both SMA samples (SMA_A and SMA_B) lacked *SMN1* and contained 2 copies of *SMN2* (Fig 1A). Both healthy cell lines (Healthy_A and Healthy_B) had two copies of *SMN1*, and varied in their *SMN2* copy numbers with Healthy_A myoblasts having 2 *SMN2* copies and Healthy_B cells had only 1 *SMN2* copy (Fig 1A). We measured full-length *SMN2* (FL) and *SMN2* lacking exon 7 (Δ7) mRNA levels in the 4 test cell lines by qRT-PCR. The levels of both *FL* and *Δ7* mRNA as well as the *FL/Δ7* ratio were significantly reduced in SMA myoblasts (Fig 1B). Immunoblot analysis revealed a significant reduction of SMN protein in SMA myoblasts to less than 30% compared to the levels in healthy cells (Fig 1C). This pattern of reduced SMN protein expression in hESC derived SMA cell lines remained stable over three days myogenic differentiation (Fig 1D). Taken together, the molecular phenotype of hESC derived SMA myoblasts confirmed all molecular characteristics of cells that might be associated with the disease state of SMA Type 1.

### Altered myogenic marker expression in myoblasts from SMA patients

**Fig 2:**
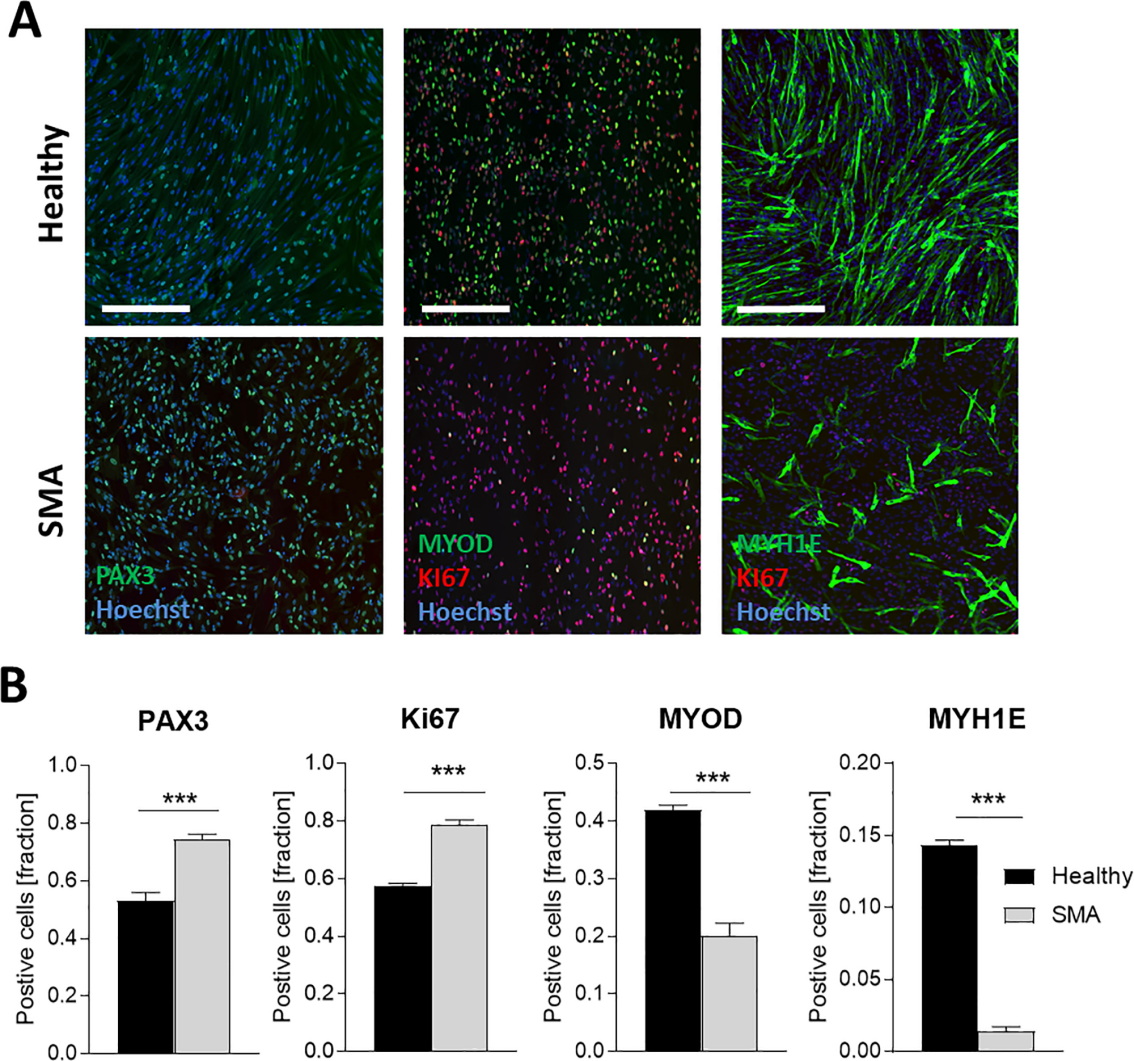
Altered myogenic marker expression in myoblasts from SMA patients. Representative fluorescence microscopy showing myoblasts stained for PAX3, MYOD, Ki67 and MYH1E as well as nuclei (Hoechst) (A). Quantification of PAX3, Ki67, MYOD, MYH1E positive cells showed significant increase of early myogenic markers (PAX3) and proliferation marker (Ki67) and reduced late myogenic markers (MYOD and MYH1E) in SMA Type 1 cells (B). Data represented as mean ± SEM of n = 6 individual assessment. ***: p < 0.001, two-tailed unpaired Student's t-test. Scale bar: 300μm.

**Fig 3:**
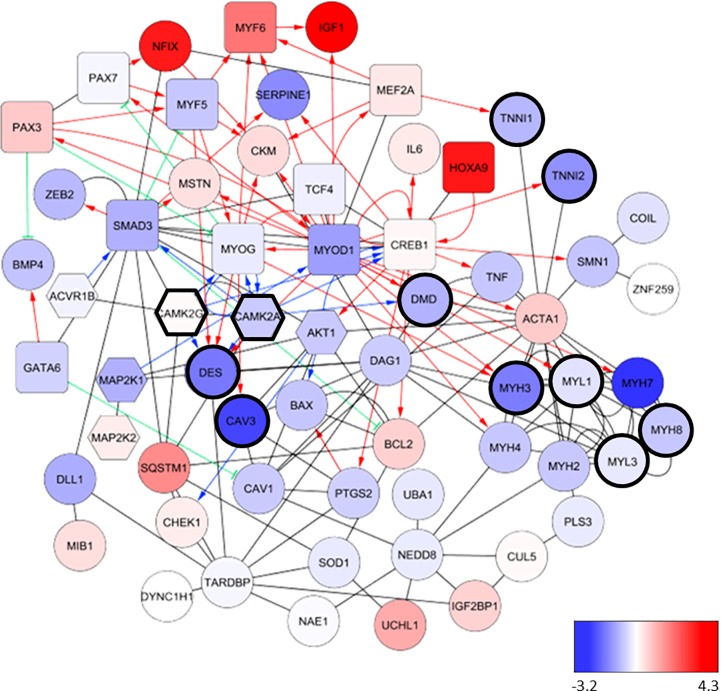
Alterations in the transcripts of myoblasts from SMA patients. Genes from the NanoString assay panel were analyzed for direct pairwise interactions using MetaBase (www.metabase.com; ubiquitination and cleavage events were excluded). The constructed interaction network shown here includes “binding” (black lines), “positive transcription regulation” (red lines with arrow tips), “negative transcription regulation” (green lines with “T” arrows), and “phosphorylation” (blue lines with arrow tips). Nodes not connected to this major cluster were excluded. Nodes are colored by the log fold change values between healthy and diseased–blue means down in disease, red means up (Table 1). Transcription factors are depicted as rounded rectangles, and kinases as hexagons. All other nodes are circles. Eleven nodes that belong to the “Muscle_contraction” network from REACTOME are highlighted by a thicker node line. This network is significantly down-regulated as a whole. Graph generated using CytoScape (www.cytoscape.org). Please note that the assay used for the detection of *SMN* gene expression detects both *SMN1* and *SMN2*. Hence, the origin of an expression signal can only be made in a context where one of the two genes is missing.

After confirming the phenotype of or hESC derived SMA myoblasts we then examined markers of myogenic precursors (PAX3), myoblasts and myocytes (MYOD, MYH1E), and cellular proliferation (Ki67). SMA myoblasts expressed PAX3 and the Ki67 at a significantly higher level compared to the healthy cells (Fig 2A and 2B). Additionally, a greater fraction of Healthy_A and Healthy_B myoblasts expressed MYOD and MYH1E as compared to the SMA myoblasts (Fig 2A and 2B). The data suggested a delay of the developmental status of the SMA myoblasts and prompted us to further evaluate global alterations in these cells. A NanoString array was utilized to assay expression of 71 genes involved in skeletal muscle differentiation and function. We found a total of 32 such over-represented networks (p value cutoff 0.001), mostly from the REACTOME database and the NCI Nature collection. For these networks, we consider the NanoString gene set to be informative. We ranked all of them based on Wilcoxon test using the log fold changes between healthy and patient cells (Table 1). Of the 32 analyzed networks, only two, very closely related networks (“Muscle_contraction” and “Striated_muscle_contraction”) were affected at a significance level of 0.001. We did not find any networks to be significantly down-regulated in healthy compared to patient cells, but the REACTOME “Muscle_contraction” network was strongly significantly down-regulated in patient cells compared to controls (Fig 3). Eleven out of its 197 member genes, highlighted in Fig 3 and in Table 1, were present in the NanoString data set. Reduction of *MYOD* and increased *PAX3* transcripts were confirmed in SMA myoblasts, in parallel with protein expression assayed by immunofluorescence. In alignment with the hypothesized deregulation of early and late differentiation markers, we observed that markers expressed during early embryonic and myogenic development such as *HOXA9* and *PAX3* were elevated in SMA myoblasts, in contrast to transcripts such as *MYOD*, *TNNI1*, *TNNI2*, *DES* and *MYOG* which were reduced in SMA myoblasts. All proteins of the myosin family analyzed in this experiment, ranging from the embryonic expressed *MYH3*, *MYH8*, *MYH2* (Type 2A muscle fibers), fetal *MYL3*, *MYH7* (Type 1 muscle fibers), and *MYL1* (fast muscles) to postnatal *MYH4* were decreased in SMA myoblasts (Fig 3, Table 1).

Pathway network analysis with the 71 genes showed clusters of transcripts known to be involved in muscle contraction were significantly downregulated, namely *MYH3*, *MYH8*, *MYL3*, *TNNI1*, *TNNI2*, *DES*, *CAV3*, *DMD*, *CAMK2A* and *CAMK2G* (highlighted in Fig 3). Taken together, the data suggests a profound alteration in the myogenic differentiation and myogenic function in SMA myoblasts.

### Impaired myogenic differentiation in SMA myotubes

**Fig 4:**
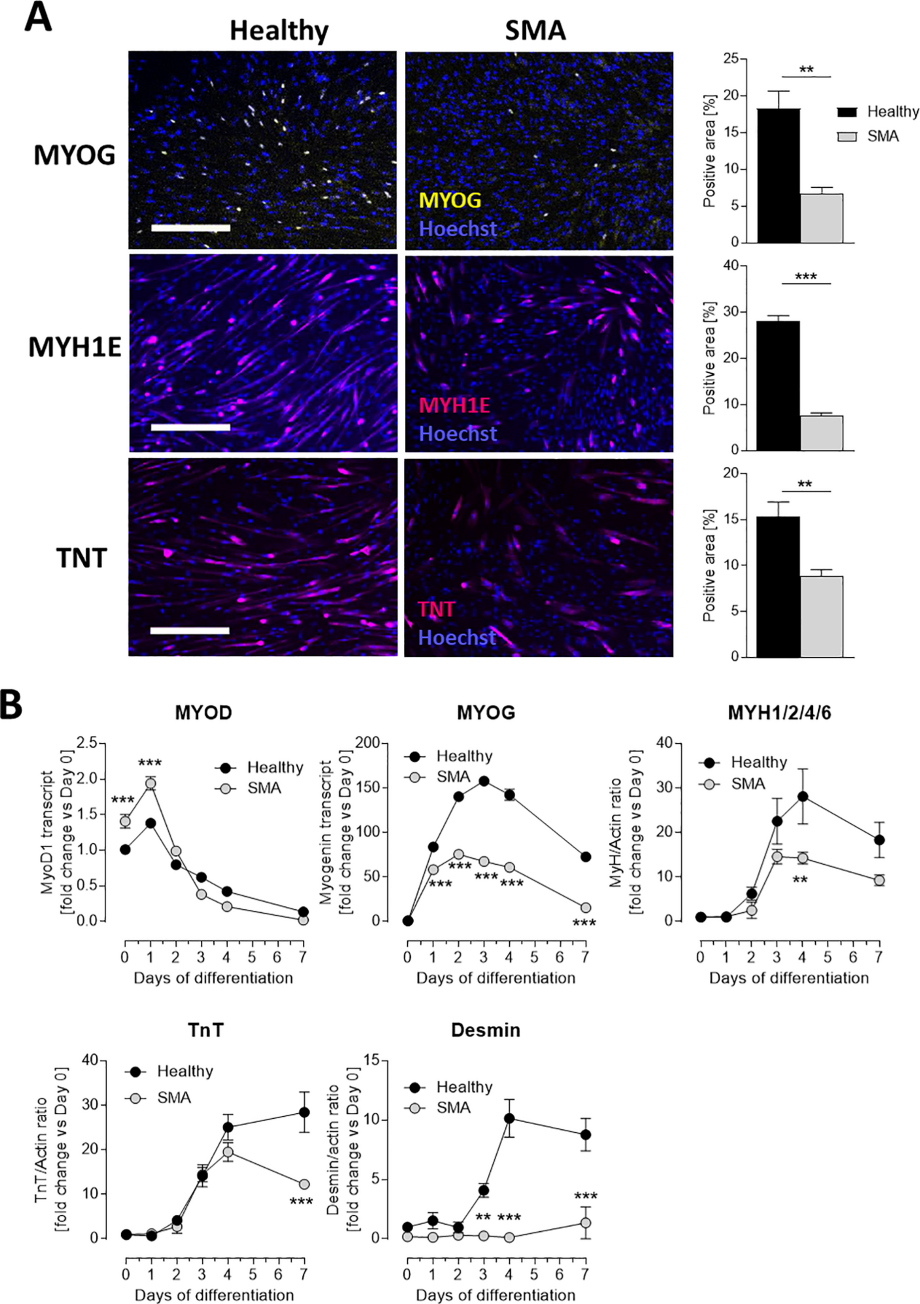
Impaired myogenic differentiation in myotubes from SMA patients. Representative fluorescence microscopy showing myotubes stained for MYOG, MYH1E and TNNT as well as cell nuclei (Hoechst) and quantification of the positively stained cells showing significant decrease of the later developmental markers in SMA Type 1 cells (A). Altered transcription and translation of myogenic factors (*MYOD*, *MYOG* analyzed by RT-qPCR; MYH1/2/4/6, TNNT and Desmin analyzed by Western Blot) during 7 days of culturing observed in SMA Type 1 cells (B). Data represented as mean ± SEM of n = 6 individual samples and normalized to unaffected day 0. **: p < 0.01; ***: p < 0.001, two-tailed unpaired Student's t-test. Scale bar: 350μm.

To verify if the observed alterations in expression levels resulted in a change in myogenic potential, we differentiated the myoblasts for three days in myotube formation medium and stained the cells for late stage myogenic markers. SMA myotubes had significantly reduced cells positive for MYOG, MYH1E and TNNT (Fig 4A). To characterize myogenic maturation in greater detail we performed a time course analysis of the MYOD and MYOG mRNA expression levels as well as MYH, DES, and TNNT protein levels in healthy and SMA cells over 7 days of differentiation (Fig 4B). At day 0 and day 1 of differentiation unaffected cells expressed higher levels of MYOD than SMA cells. However, a rapid decrease in MYOD expression was observed in both cell types over the remaining time course. In contrast, levels of *MYH*, *TNNT* and DES (markers of late stage myogenesis) increased after 3 days (Fig 4B) in in healthy cells. In contrast, upregulation of DES was entirely lost in SMA cells during differentiation (Fig 4B). Overall, these analyses were in concordance with the NanoString results (Fig 3, Table 1).

### Cholinergic Ca^2+^ response is reduced in SMA myotubes

**Fig 5:**
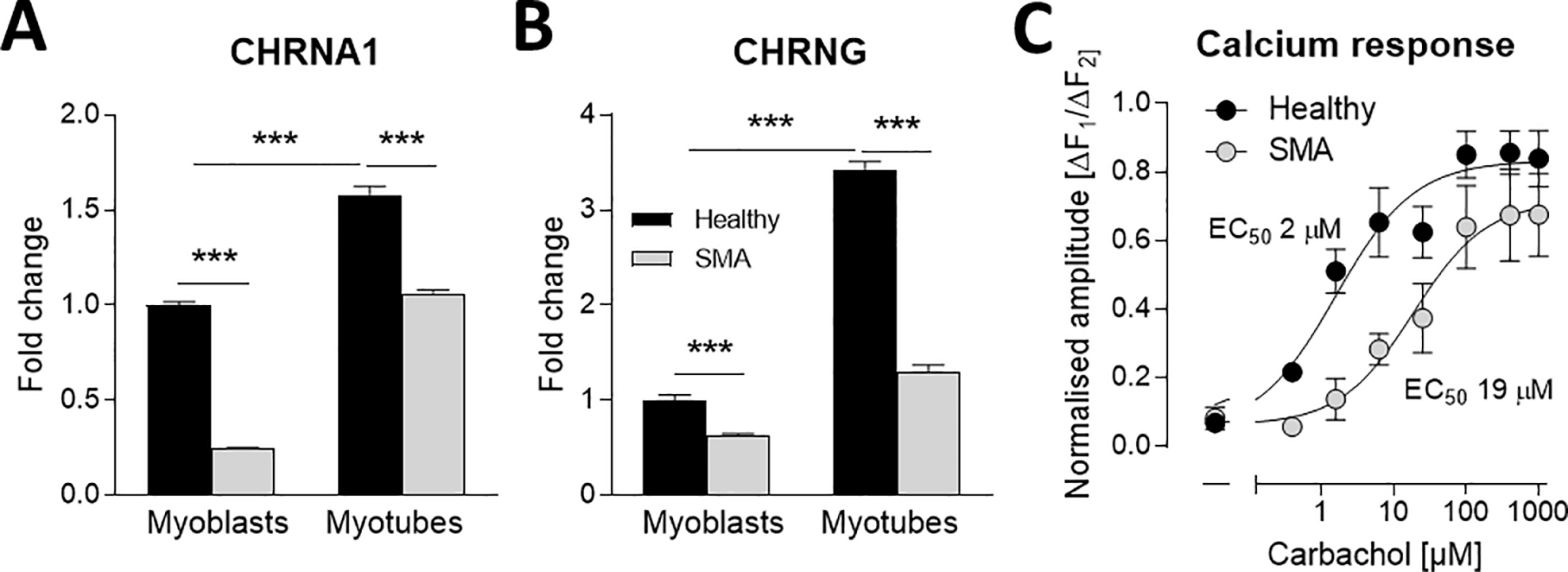
Reduced cholinergic Ca2+ response in differentiated myotubes from SMA patients. RT-qPCR of relative AChR subunit expression CHRNA1 (A) and CHRNG (B) decreased in SMA Type 1 cells. Fluorometric measurement of intracellular Ca2+ concentrations changes by carbachol treatment in ESC-SkM. Dose-response curve carbachol responses for WT and SMA ESC-SkM showed different sensitivity between genotypes (EC50 values, p < 0:05, sum-of-squares F-test). Baseline-subtracted amplitudes of carbachol responses normalized to baseline subtracted amplitudes of 4-Br-A23187 responses. These ratios were normalized to maximal values within an experiment. Data represented as mean ± SEM (A, B) and mean ± SEM of n = 6–8 individual samples from two independent experiments. *: p < 0:05, **: p < 0:01, ***: p < 0:001, two-tailed unpaired Student's t-test.

Delayed myogenesis is expected to result in less mature myotubes, and we aimed next to confirm this functionally by induction of a cellular Ca^2+^ change in response to cholinergic stimulation by carbachol treatment. We first measured the transcriptional expression of alpha (*CHRNA1*) and gamma (*CHRNG*) subunits of the embryonically expressed nicotine receptor. Expression levels of both subunits increased during myogenic differentiation, however at a statistically higher level in healthy as compared to SMA cells. (Fig 5A and 5B). To identify if this transcriptional reduction resulted in a functional deficit on carbachol-induced Ca^2+^ influx, we measured intracellular Ca^2+^ concentrations in differentiated myotube cultures (Fig 5C). Both myotube cultures from healthy donors and SMA patients showed a dose-dependent response to carbachol treatment. However, healthy myotubes were more sensitive to carbachol treatment than SMA cells as indicated by an EC_50_ value of 2 μM in healthy cells and 19 μM in SMA cells (Fig 5C), well in line with the reduced subunit expression observed in SMA myotubes. The data suggests that reduced nicotinic acetylcholine receptor expression results in a reduced functional Ca^2+^ response to carbachol stimulation in SMA myotubes.

### Impaired glycolytic and metabolic function in SMA myoblasts

**Fig 6:**
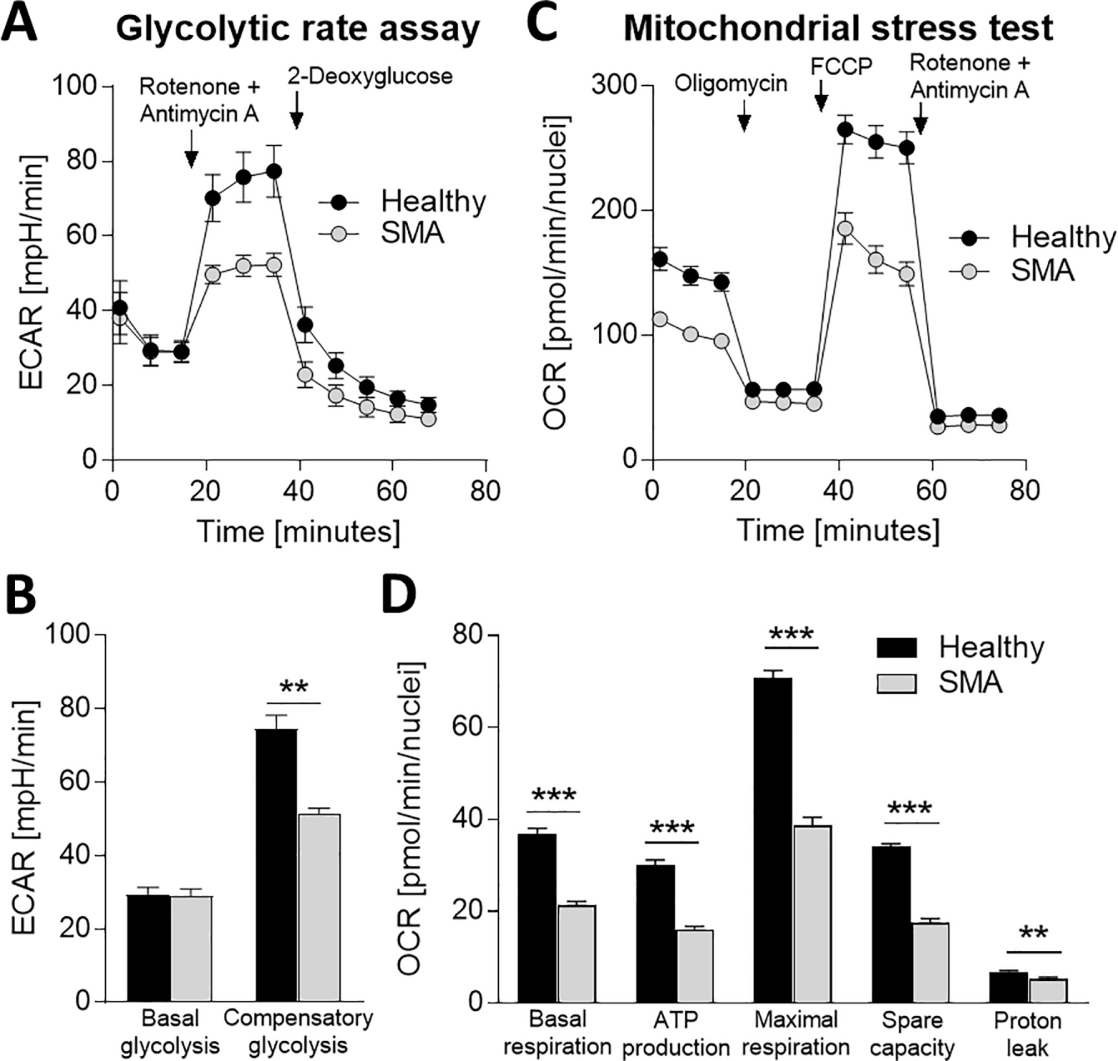
Impaired glycolytic function and metabolic activity in myoblasts from SMA patients. Analysis of glycolysis with the glycolysis rate assay using the Seahorse revealed decreased levels of compensatory glycolysis in SMA Type 1 myoblasts (A, B). Mitochondrial stress test detected impaired oxidative phosphorylation in SMA Type 1 myoblasts ranging from reduced levels during basal respiration, ATP production, maximal respiration to spare capacity and proton leak (C, D). ECAR: extracellular acidification rate, OCR: oxidative consumption rate, Data represented as mean ± SEM of n = 3 individual samples. *: p < 0:05, **: p < 0:01, ***: p < 0:001, two-tailed unpaired Student's t-test.

Muscle cells necessarily require high energy, and impairments in essential (glycolytic and oxidative) energy pathways impact muscle functionality. To investigate if biological processes such as glycolytic and mitochondrial function are affected in SMA myoblasts and myotubes, we analyzed the glycolytic rate and mitochondrial respiration using the Seahorse XF instrument. The glycolysis rate was measured as extracellular acidification rate (ECAR) and the oxygen consumption rate (OCR) was analyzed to monitor mitochondrial stress (Fig 6). Basal glycolysis was unchanged between healthy and SMA myoblasts, however compensatory glycolysis after Rotenone/Antimycin A treatment was significantly reduced in SMA myoblasts compared to health controls (Fig 6A and 6B). Furthermore, significant deficits in mitochondrial basal respiration, ATP production, maximal respiration, spare respiratory capacity and proton leak were observed in SMA myoblasts (Fig 6C and 6D). In summary, SMA myoblasts depict reduced glycolysis and mitochondrial respiration.

### Reduced ATP production in SMA generated myotubes

**Fig 7:**
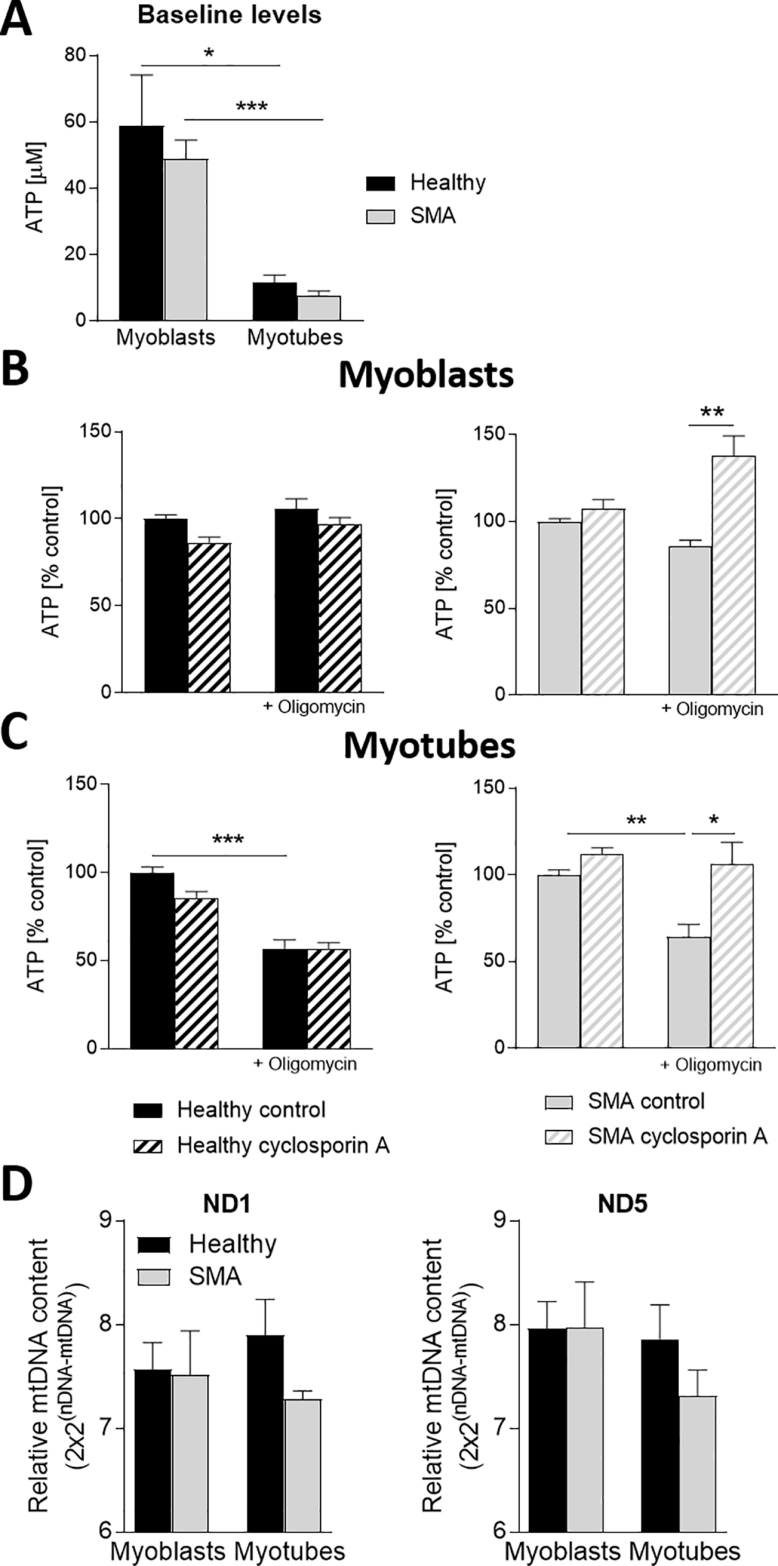
Reduced ATP production in myotubes from SMA patients. ATP levels were reduced in myotubes of SMA and unaffected cells compared to myoblasts (A). Challenge with Oligomycin and treatment with Cyclosporin A in myoblasts increased ATP levels only in SMA Type 1 derived myoblasts (B), while in myotubes Oligomycin challenge decreased in all cells the ATP levels, but also here Cyclosporin A had only a protective effect on SMA Type 1 cells (C). Mitochondrial DNA levels, an indicator for the number of mitochondrial, were not significantly reduced in SMA derived cells (D). Data represented as mean ± SEM of n = 3 individual samples. p < 0:05, **: p < 0:01, ***: p < 0:001, two-tailed unpaired Student's t-test, (A-D n = 3).

Having evidence that myoblasts from SMA patients have impaired energy generation, we aimed to confirm this important aspect by measuring ATP levels in healthy and SMA myoblasts and myotubes. We further investigated if the oxidative phosphorylation was also affected in more mature myotubes and if ATP production could be improved by treatment with Cyclosporin A, a compound known to increase mitochondrial capacity in skeletal muscle cells [40]. Baseline ATP levels in healthy and SMA myoblasts and myotubes were not significantly altered (Fig 7A). However, ATP levels in both healthy and SMA myotubes were significantly lower than in myoblasts, although mitochondrial DNA of ND1 and ND5 indicated similar total mitochondria numbers in myoblasts and myotubes (Fig 7B). After treatment with Oligomycin, ATP production was unchanged in healthy myoblasts and only mildly reduced in SMA myoblasts, but strongly reduced in both healthy and SMA myotubes (Fig 7C and 7D). Importantly, Cyclosporin A pre-treatment was capable of increasing ATP production after Oligomycin challenge in SMA myoblasts and myotubes but was without effect in healthy cells. Myotubes are known to depend in their energy consumption much more on mitochondrial oxidative phosphorylation than myoblasts [41]. This is a likely explanation for the lack of oligomycin and cyclosporin effects on healthy myoblasts which produce ATP mainly via glycolysis. In contrast, myotubes require more energy and an ATP synthesis through oxidative phosphorylation in mitochondria. The SMA phenotype, however, apparently creates an intrinsic energy deficit that requires them to use mitochondrial ATP production thus makes them sensitive to oligomycin and cyclosporin effects. It will be of great interest to explore the SMA energy deficit in myogenic cells. In addition to the identification of an SMA-specific energy deficit phenotype in these cells that was ameliorated by mitochondrial protective compounds we also validated the differentiation protocol of the myoblasts and myotubes by monitoring their different energy state.

## Discussion

SMN expression is ubiquitous, phylogenetically conserved, and required for normal lifespan across species. Although there is a large body of literature describing the loss of SMN in motor neurons and neuromuscular junctions, the mechanisms underlying the pathophysiological features of SMA in peripheral tissues such as muscle are less clear. Animal models and systems of cellular differentiation have demonstrated that tightly controlled levels of SMN are necessary for normal skeletal muscle differentiation and maturation [24,26,37]. To investigate a possible altered regulation of myogenesis in SMA we recapitulated muscle development *in vitro* using hESC derived skeletal muscle. This approach allowed for the examination of phenotypes in isolated myogenic cells without the confounding contribution of degenerating motor neurons.

In this study we demonstrated that the myogenic cells derived from SMA patient hESCs recapitulate the molecular features of Type 1 SMA and displayed major deficits not only in myogenic development but also in other biological processes including cholinergic signaling, glycolysis, and oxidative phosphorylation. Here we described a novel model for generating myoblasts and myotubes for interrogating the cell autonomous effects of SMN deficiency during muscular development. Previous work by Hosoyama et. al (2014) described a human pluripotent stem cell derived myogenic differentiation protocol using sphere-based culture (EZ sphere) which was used to generate skeletal muscle from SMA iPSCs. The authors of this study did not observe any obvious phenotypic differences in the myotubes generated from SMA patient-specific lines, however they noted that some variations were observed in the number of myogenic progenitors and mature myotubes between the lines [42]. However, the Hosoyama protocol is not amenable to studying cell autonomous, muscle specific defects given the fact that EZ spheres cultures contain a mixed population of neural (~50% Nestin positive) and myogenic progenitors [42]. In contrast, the human SMA myogenic cells described here represent a nearly homogenous myogenic culture that is suitable for screening and investigating compounds that therapeutically target mitochondrial respiration in muscle, calcium signaling in muscle or target myogenic differentiation processes.

Muscle cells in cultures from severe SMA Type 1 patients have previously been shown to manifest defective development and disorganized myotubes (46,47). Here we demonstrate that defects in myogenic differentiation of skeletal muscle cells develop regardless of their previous contact with SMN deficient motor neurons. This strongly suggests an autonomous, muscle cell-specific role of SMN that impacts both myogenic development and metabolic function. Furthermore, muscular developmental defects may contribute, at least in part, to the neuromuscular pathology observed in SMA and suggest that therapeutic interventions for SMA are to be employed at early neonatal stages to address deregulated signaling in neuromuscular junction.

Numerous publications describe the neuromuscular deficits in SMA as ‘secondary’ effects to the loss or dysfunction of motor neurons and subsequent denervation [32,43–45]. To amend this view, we described a dysregulated myogenic differentiation and deficits in myogenic maturation illustrated by functional changes in Ca^2+^ responsiveness and metabolic activity. In particular, we speculate that the lack of mature cholinergic receptors in SMA myoblasts and myotubes could play a significant role in destabilizing the neuromuscular junction. To complement this point, the dysregulation of glycolysis, oxidative phosphorylation and mitochondrial respiration show dramatic impairment in SMA myoblasts. These muscle abnormalities can be regarded as underlying pathogenic events which could play a negative role on the normal function of the neuromuscular junction.

## Conclusion

There is mounting evidence supporting a direct role for SMN in peripheral organs and especially in the skeletal muscles as a direct contributor to SMA. Addressing multiple aspects of muscle pathophysiology in SMA patients may be an important synergistic strategy to improve the lives of SMA patients. Thus, restoration of SMN levels beyond the CNS by intrathecal delivery of SMN targeted therapies, may be required to completely rescue the disease phenotype [46,47]. Due to likely early developmental defects caused by loss of SMN1 gene function, postnatal restoration of functional SMN levels in patients may not be sufficient to ameliorate all disease symptoms [47,48]. Despite SMN-targeted therapies, patients might later show neuromuscular weaknesses due to a reduced functional reserve [46,47] To this end, using hESC-derived myogenic cells we have described several diverse SMA phenotypes that could be immediately amenable to both screening and identification of skeletal muscle-specific therapeutics for SMA.
